# Rational diagnostic strategies for Lyme borreliosis in children and adolescents: recommendations by the Committee for Infectious Diseases and Vaccinations of the German Academy for Pediatrics and Adolescent Health

**DOI:** 10.1007/s00431-012-1779-4

**Published:** 2012-07-11

**Authors:** H. I. Huppertz, P. Bartmann, U. Heininger, V. Fingerle, M. Kinet, R. Klein, G. C. Korenke, H. J. Nentwich

**Affiliations:** 1 Schwachhauser Heerstr. 163a, 28211 Bremen, Germany; 2 Department of Neonatology, University Bonn, 53113 Bonn, Germany; 3 University Children’s Hospital Basel (UKBB), Spitalstrasse 33, 4056 Basel, Switzerland; 4 Bayerisches Landesamt für Gesundheit und Lebensmittelsicherheit, Veterinärstr. 2, 85764 Oberschleißheim, Germany; 5 Graf-Luckner-Str. 4f, 24159 Kiel, Germany; 6 Ministerium für Gesundheit und Verbraucherschutz, Referat B1, Ursulinenstraße 8-16, 66111 Saarbrücken, Germany; 7 Neuropädiatrie, Elisabeth-Kinderkrankenhaus, Rahel-Straus-Straße 10, 26133 Oldenburg, Germany; 8 Deutsche Akademie für Kinder- und Jugendmedizin e.V., Chausseestr. 128/129, 10115 Berlin, Germany

**Keywords:** Lyme borreliosis, *Borrelia burgdorferi*, Neuroborreliosis, Lyme arthritis, Diagnosis, Children, Recommendations

## Abstract

The varying clinical manifestations of Lyme borreliosis, transmitted by *Ixodes ricinus* and caused by *Borrelia burgdorferi*, frequently pose diagnostic problems. Diagnostic strategies vary between early and late disease manifestations and usually include serological methods. Erythema migrans is pathognomonic and does not require any further laboratory investigations. In contrast, the diagnosis of neuroborreliosis requires the assessment of serum and cerebrospinal fluid. Lyme arthritis is diagnosed in the presence of newly recognized arthritis and high-titer serum IgG antibodies against *B*. *burgdorferi*. The committee concludes the following recommendations: Borrelial serology should only be ordered in case of well-founded clinical suspicion for Lyme borreliosis, i.e., manifestations compatible with the diagnosis. Tests for borrelial genomic sequences in ticks or lymphocyte proliferation assays should not be ordered. When results of such tests or of serological investigations that were not indicated are available, they should not influence therapeutic decisions. Laboratories should be cautious when interpreting results of serological tests and abstain from giving therapeutic recommendations and from proposing retesting after some time without intimate knowledge of patient's history and disease manifestations.

## Introduction

Lyme borreliosis is caused by infection with *Borrelia burgdorferi* sensu lato and transmitted by the bite of the tick *Ixodes ricinus* [26]. In Europe, Lyme borreliosis may present as a variety of manifestations that are classified into early and late manifestations in children and adolescents [12] (Table 1). The correct diagnosis is made or is at least strongly suggested by history and physical examination. Borrelia-specific laboratory results confirm the clinical suspicion [31]. Besides the direct methods for detecting the infectious agents in body fluids or tissues, for example, by polymerase chain reaction (PCR), serological methods are usually used on blood and cerebrospinal fluid. Primarily, enzyme immunoassays, distinguishing between immunoglobulin M and G antibodies, are applied as screening tests. Because of their low specificity, (line) immunoblot assays, often with recombinant antigens, are used as confirmatory assays in case of a reactive (positive or borderline) enzyme immunoassay [7]. A few antigens are especially helpful including VlsE and OspC for early disease and p83/100 for late borreliosis. By comparing antibody concentrations in serum and cerebrospinal fluid, it is possible to detect intrathecal antibody production [18].

**Table 1:**
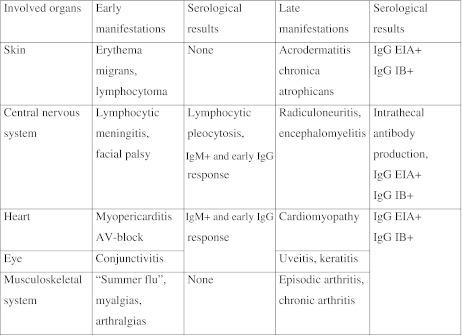
Manifestations of Lyme borreliosis in children and adolescents (modified from Huppertz [12])

Early manifestations can be observed after some days up to a few weeks after infection and are self-limiting. Assessment of antibodies in serum may still be unremarkable or show an early seroconversion including IgM antibodies and low-titer IgG antibodies against *B*. *burgdorferi*. Late manifestations show up months to years after infection, may become chronic and, in rare cases, lead to lasting organ damage. Serological results show high-titer IgG antibodies against *B*. *burgdorferi*; IgM antibodies may persist. *EIA* enzyme immunoassay, *IB* immunoblot

These diagnostic strategies were established more than 10 years ago, and scientific progress, since then, is minimal. Although there are good review articles, for example, in the handbook of the German Society for Pediatric Infectious Diseases [21], diagnosing Lyme borreliosis continues to be difficult, and therefore, a rational strategy is presented here for everyday use. The diagnostic strategies discussed here apply to European, but not necessarily to North American Lyme borreliosis.

## Erythema migrans

Erythema migrans is by far the most frequent manifestation of Lyme borreliosis in Europe, amounting to nearly 90 % of all cases [13]. Clinical presentation of erythema migrans is pathognomonic, and therefore, usually no laboratory tests are necessary for diagnosis [26]. Because serology is frequently negative at this stage, antibodies should not be determined. In rare cases of atypical erythema migrans, clinical diagnosis may be difficult. In these cases, the present extent of the erythema may be marked with a pen and should be reexamined 1–2 days later. If the erythema has expanded, the diagnosis of erythema migrans is most likely correct. PCR of skin biopsy is a good test to confirm a diagnosis of erythema migrans, and results are truly positive in about 70 % of cases when good biopsy and laboratory techniques are used [3]. However, skin biopsy is seldom justified. Response to antibiotic treatment is only confirmed by disappearance of erythema migrans and not by serological means.

## Other manifestations

When other manifestations of Lyme borreliosis are suspected, and erythema migrans is not present, serological assays should be performed [31].

## Suspicion of early neuroborreliosis

Lyme borreliosis should be considered in patients with cranial nerve palsy, in particular, seventh nerve palsy, and signs of meningitis with or without headache, lethargy, or irritability for about 9 days on average before admission [25, 27]. Stiffness of the neck is often very mild or absent and must be searched for carefully. In contrast, patients with aseptic/viral meningitis usually display obvious nuchal rigidity and have a history of less than 6 days, often 1 or 2 days only [4, 27]. Especially in the absence of cranial nerve palsy, the possible diagnosis of Lyme borreliosis is not being considered often enough. Patients with headache as a sole manifestation usually do not have neuroborreliosis. In comparison to patients with noninflammatory headaches of frequent and various causes, the headaches of patients with neuroborreliosis usually have a clearly indicated beginning and a short duration of less than a month [27]. To confirm neuroborreliosis, antibodies against *Borrelia burgdorferi* are assessed in serum and cerebrospinal fluid. In the case of early neuroborreliosis, there is lymphocytic pleocytosis in the cerebrospinal fluid, but often, intrathecal antibody production cannot yet be found [24]. If cerebrospinal fluid yields pleocytosis with ≥90 % mononuclear cells, there are no other remaining causes apart from tuberculous meningitis. In very early stages of the disease, serological results in serum may still be negative [4]. In typical cases, antibodies of the immunoglobulin IgM class against *B*. *burgdorferi* are found by enzyme immunoassay and are confirmed by two or more bands by IgM immunoblot. Later, IgG antibodies may be detected by enzyme immunoassay, and the number of bands in the IgG immunoblot increases gradually. Therefore, in case of a negative serology and continuing suspicion of neuroborreliosis, it may be useful to determine antibodies in serum again 2 or 3 or 4 weeks later to find seroconversion. PCR in cerebrospinal fluid often is positive only in very early cases when there are not yet antibodies against *B*. *burgdorferi* present. Although a positive PCR supports the diagnosis of neuroborreliosis, a negative PCR does not exclude it [18]. Therefore, PCR should not be used routinely to make a diagnosis of neuroborreliosis, but only in complex cases.

## Suspicion of late neuroborreliosis

The neurological manifestations have usually been present for some time, and a number of different diagnoses have been considered, including multiple sclerosis, Guillain–Barré syndrome, pseudotumor cerebri, and cerebral vasculitis. The clinical presentation may be varying, including headache, lethargy, irritability, and focal neurological signs. Late neuroborreliosis is very rare in children.

Lymphocytic pleocytosis is not necessarily found in cerebrospinal fluid, since it may occur intermittently. However, there is borrelia-specific intrathecal antibody production, usually of immunoglobulin G [18]. In addition, there are intrathecal oligoclonal bands and a high protein concentration in cerebrospinal fluid. In serum, there is a positive enzyme immunoassay for IgG antibodies against *B*. *burgdorferi*, confirmed by IgG immunoblot with a multitude of bands.

## Suspicion of Lyme arthritis

In the case of newly appearing arthritis, a borrelial serology should be obtained, especially in mono- or oligoarthritis, including the knee joint [14]. In case of Lyme arthritis, the enzyme immunoassay is highly positive for IgG antibodies against *B*. *burgdorferi*; the results of which are confirmed by immunoblot with a multitude of bands. PCR in synovial fluid may be positive. The rate of correctly positive results by PCR may be increased by using synovial tissue. However, the suspicion of Lyme arthritis is not sufficient justification for performing a synovial biopsy, and laboratory confirmation of the diagnosis primarily relies on serum antibody determination.

## Suspicion of further manifestations

There is a variety of further rare manifestations of Lyme borreliosis. When there is a reasonable suspicion of Lyme borreliosis, diagnosis is supported by serology. The expected serological results vary with the stage of the disease, i.e., if the patient has an early or late manifestation of Lyme borreliosis. Eyes may be involved by keratitis, iridocyclitis, or uveitis intermedia. Cardiac involvement may appear as AV block or carditis. Acrodermatitis chronica atrophicans is a late skin manifestation of borreliosis and very rare in children. In borrelial lymphocytoma, usually found at earlobes, nipples, or testicular sacks, serology is not infrequently false negative. Therefore, diagnosis may be established exclusively by clinical means, as is the case for erythema migrans. The issue of summer flu, including fever, joint pain, and fatigue without signs of mucous membrane involvement is still unclear, although it may be a manifestation of early Lyme borreliosis. The clinical presentation is too varied, and serological examinations often are still negative, or false positive serological results lead to misdiagnosis and unnecessary treatment [23]. The disease usually is self-limiting after a few days.

## Mental state disturbances and vague somatic symptoms (false manifestations of Lyme borreliosis)

Borreliosis is sometimes suspected in patients with functional problems or mental state disturbances. However, symptoms usually do not fit with known manifestations of Lyme borreliosis [9].

In case of chronic headache or diminishing academic achievements, often patients are not able to indicate when complaints started, which is not typical of neuroborreliosis. If suspicion of neuroborreliosis remains, lumbar puncture should be performed, which excludes neuroborreliosis if findings are normal, i.e., absence of pleocytosis and intrathecal antibody production. If antibody determination is only being done in serum, the result is without relevance for the cause of headaches, and frequently, serological results cannot be interpreted in the absence of results from cerebrospinal fluid [2].

In case of muscular and skeletal complaints, sometimes Lyme arthritis is suspected, although arthritis is missing, a sign which is necessary for the diagnosis of Lyme arthritis. If serology has been performed in spite of the absence of arthritis, the lack of antibodies of immunoglobulin G against *B*. *burgdorferi* excludes Lyme arthritis. If this determination of specific IgG antibodies is positive, anti-*B*. *burgdorferi* antibody production has been detected; however, the cause of musculoskeletal pain remains unclear in the absence of arthritis. This is due to the fact that the positive predictive value is low if the prevalence of the tested trait is low [23]. Moreover, when assays for antibodies against *B*. *burgdorferi* are ordered in patients with an absence of objective signs of manifestations compatible with Lyme borreliosis, serology often cannot be interpreted. Therefore, in case of nonspecific complaints like headache, limb pain, or mental state disturbance, borrelial serology should not be performed.

## Recommendations by laboratories concerning the interpretations of serological results

The interpretation of results of serology for *B*. *burgdorferi* may be difficult and sometimes is indeterminate. Interpretation should always be done in conjunction with complete clinical knowledge of the patient.

Some laboratories recommend that borrelial serology be repeated after a few weeks or months. However, this only rarely leads to new evidence. An exception from this rule is early disseminated disease, including early neuroborreliosis. Therefore, serology should be repeated after an interval of time only in very few well-founded cases.

Sometimes the interpretation of serology results by laboratories contains recommendations for treatment. However, this would require complete knowledge of clinical presentation and previous therapeutic measures. As these are usually not available in the laboratory, these recommendations often are inaccurate or wrong.

In addition to these, other diagnostic strategies have been recommended: The analysis of ticks which have been removed from human skin for the presence of borrelia, and the assessment of the ability of lymphocytes from peripheral blood to proliferate after addition of borrelial antigens.

## Analysis for borrelia of ticks which have been removed from human skin

Some laboratories recommend and advertise assessing ticks by PCR for borrelial genomic sequences when they have been removed from the skin of a human host. Laboratories report the presence of borrelia, sometimes also the genotype and the number of copies. In case of a positive result, these laboratories recommend either antibiotic prophylaxis or serological assessment immediately and a few weeks later to detect seroconversion.

There are no convincing data, justifying the use of these tests in ticks removed from humans or patients. There are no data presented for false positive results. It is not known why the number of copies is relevant, since the minimal infectious dose of *B*. *burgdorferi* is not known [6]. If the genotype is not indicated, nonpathogenic genotypes may be included under the general term *B*. *burgdorferi* sensu lato, thus exaggerating the potential risk of transmission. In a study from Switzerland, a positive test result finding borrelia in removed ticks was not associated with the development of Lyme borreliosis [19].

The assessment of ticks for borrelial genomic sequences overestimates the importance of a single tick bite, since most tick bites are not recognized by the host, and most patients with Lyme borreliosis do not remember having been bitten by a tick [11]. When people recognize a tick on their skin and remove it early (i.e., within 24 h), most of the time, borrelia is not transmitted [30]. The risk of transmission after a tick bite is given as 4 % [8]. In a Swiss study, seroconversion occurred in 4.5 % of tick bites [18]. Most infections take an uneventful course without clinical manifestations [19]. Asymptomatic seroconversion and clinical infection are low in spite of a high percentage of infection by *B*. *burgdorferi* in ticks removed from these humans [11]. Up to 5 % of healthy blood donors display IgG antibodies against *B*. *burgdorferi* [1]; healthy forest workers, up to 52 % [20]. In German children, this rate currently is 4.8 % [22]. The apparently low transmission rate and the high proportion of asymptomatic borrelia infections after tick bites do not justify the search for borrelial genomic sequences in a removed tick. In addition, a positive result does not allow a reasonable conclusion: prophylactic antibiotic treatment after a tick bite is not recommended in Europe the more so as this treatment may not prevent infection followed by clinical manifestations [17]. Even if seroconversion is detected after a tick bite, found in paired serum assessments at an interval of 3–4 weeks, this finding is without consequences in the absence of clinical manifestation, since antibiotic treatment would only be recommended if the patient gets sick with borreliosis. In conclusion, the assessment of borrelial sequences or antigens in ticks removed from patients is without therapeutic consequence, and therefore, the test should not be performed [10].

## Assessment of the reaction of lymphocytes towards borrelia antigens

Plasma cells producing antibodies are under the control of T cells during their development. Early attempts to assess the reactivity of T cells towards borrelial antigens used the lymphocyte transformation assay. In spite of refined tests and use of recombinant antigens, it was not possible to obtain the same specificity as in serological methods including enzyme immunoassay and immunoblot [5, 15, 16]. Consequently, the lymphocyte transformation assay has not been recommended for clinical use.

Recently, there have been new attempts to introduce this test into the diagnosis of Lyme borreliosis [28, 29]. This was based on further technical improvements of the tests. In two publications, a good performance of these tests has been reported [28, 29]. Closer examination of these publications, however, shows that the patients had not been characterized, and the suspicion of the physician, sending the probe to the laboratory (“suspicion of borreliosis”), was taken as a diagnosis. Serological results were not communicated for all patients, and correct control groups were missing. Consequently, these publications are not able to contribute to the assessment of the validity of the lymphocyte transformation assay. Therefore, results of these tests cannot contribute to the management of patients.

## Conclusions of the committee

The following consequences should be drawn:

1. The committee recommends that pediatricians order borrelial serology only when there is a well-founded clinical suspicion of Lyme borreliosis following the diagnostic strategy outlined above. Patients with chronic pain, fatigue, or mental state disturbances should not be tested for Lyme borreliosis.
2. The committee recommends that insurance companies do not pay for laboratory assessments that are not indicated. This includes serological tests without well-founded clinical suspicion, tests for borrelial antigens or genomic sequences in ticks, and lymphocyte transformation assays.
3. The committee strongly recommends that laboratories that offer serological examination for Lyme borreliosis should never add therapeutic recommendations when interpreting the laboratory results. Without close knowledge of clinical manifestations and previous therapeutic measures, it is not possible to reason about therapeutic consequences of serological results.
4. In addition, the committee asks laboratories not to recommend further serological assessments after a few weeks or months when interpreting serological results. In most cases, repetition of serology does not add useful new evidence. Only the attending physician with close knowledge of history, clinical manifestations, and previous treatments may decide on the usefulness of repeating the serology after 2 or 3 or 4 weeks in a given patient.
